# Perceived Stress and Food Consumption Among Pakistani Medical Students – a Survey Study

**DOI:** 10.1192/bjo.2024.109

**Published:** 2024-08-01

**Authors:** Saad Azam, Haania Shahbaz, Fiza Azam

**Affiliations:** 1 Shaikh Khalifa Bin Zayed Al Nahyan Medical & Dental College, Lahore, Pakistan; 2 Dow Medical College, Karachi, Pakistan; 3 Dorset County Hospital, Dorchester, United Kingdom

## Abstract

**Aims:**

The aims of this study included investigating the relationship between perceived stress levels and food consumption patterns amongst Pakistani medical students. Additionally, the study meant to determine whether there is a significant difference in food choice between high-stress and low-stress groups of students. Lastly, the study aimed to identify the specific food types most commonly consumed by medical students under high stress conditions.

The investigators of this study hypothesised that there is a significant difference in food choices between high-stress and low-stress groups of medical students.

Among the common health problems reported by medical students, stress stands out as one. Factors related to educational and psychological domains result in the development of stress. Changing dietary patterns is a commonly employed strategy used to deal with stress.

**Methods:**

This study utilised an online survey administered among medical students across Pakistan. The data collection period was 4 weeks from 5th July to 5th August 2023. The survey was distributed conveniently using social media platforms. Sampling was done via the snow-ball method. Data analysis was done via SPSS.

**Results:**

Our results from the population of 138 females (68.6%) and 63 males (31.3%) concluded that there were no significant differences in the perceived stress score between genders (p-value = 0.377) and between hostelites and non-hostelites (p-value = 0.816) using the Mann–Whitney test. We found statistically significant differences in the perceived stress score among the different frequencies for the consumption of snacks (p = 0.02) and fast foods (p = 0.008), but the stress score remained non-significant for fruits and vegetables (p-value = 0.089), ready-to-eat foods (p-value = 0.134), and sweets (p-value = 0.051) with the Kruskal–Wallis test.

**Conclusion:**

While previous studies have shown a difference in perceived stress across genders and living arrangements, ours found none. In addition, we found snacks and fast foods to be the go-to for students in times of stress, but the consumption of healthier foods was not associated with a lower level of stress.

